# Supplementary material for: Association between DNA methylation and ADHD symptoms from birth to school age: a prospective meta-analysis
Source: Transl Psychiatry. 2020 Nov 12;10:398. doi: 10.1038/s41398-020-01058-z (PMC7665047; doi:10.1038/s41398-020-01058-z)

Supplementary Figures

Figure S1

Figure S1: Methylation levels of genome-wide significant CpG sites (cord blood EWAS) in brain and blood tissue of 16 participants in the BECon database.

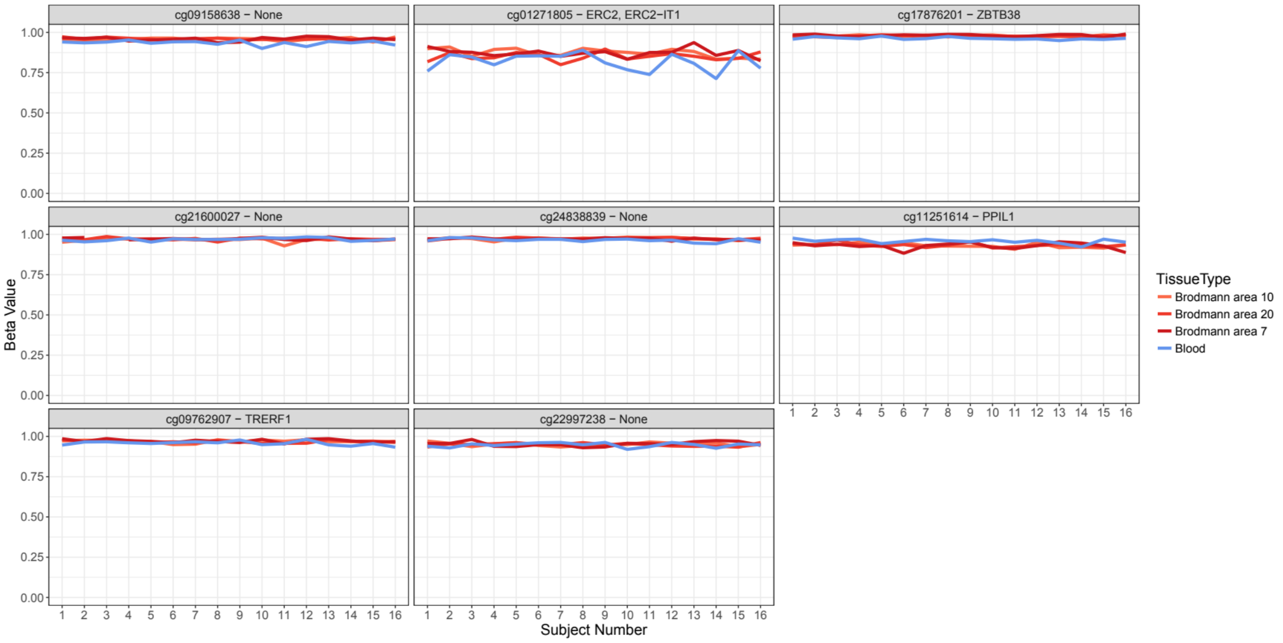

Figure S2

Figure S2: Scatterplot of genome-wide significant CpG sites (birth methylation EWAS) in the Generation R Study. Red indicates ADHD levels at age 6, green at age 8 and blue at age 10.

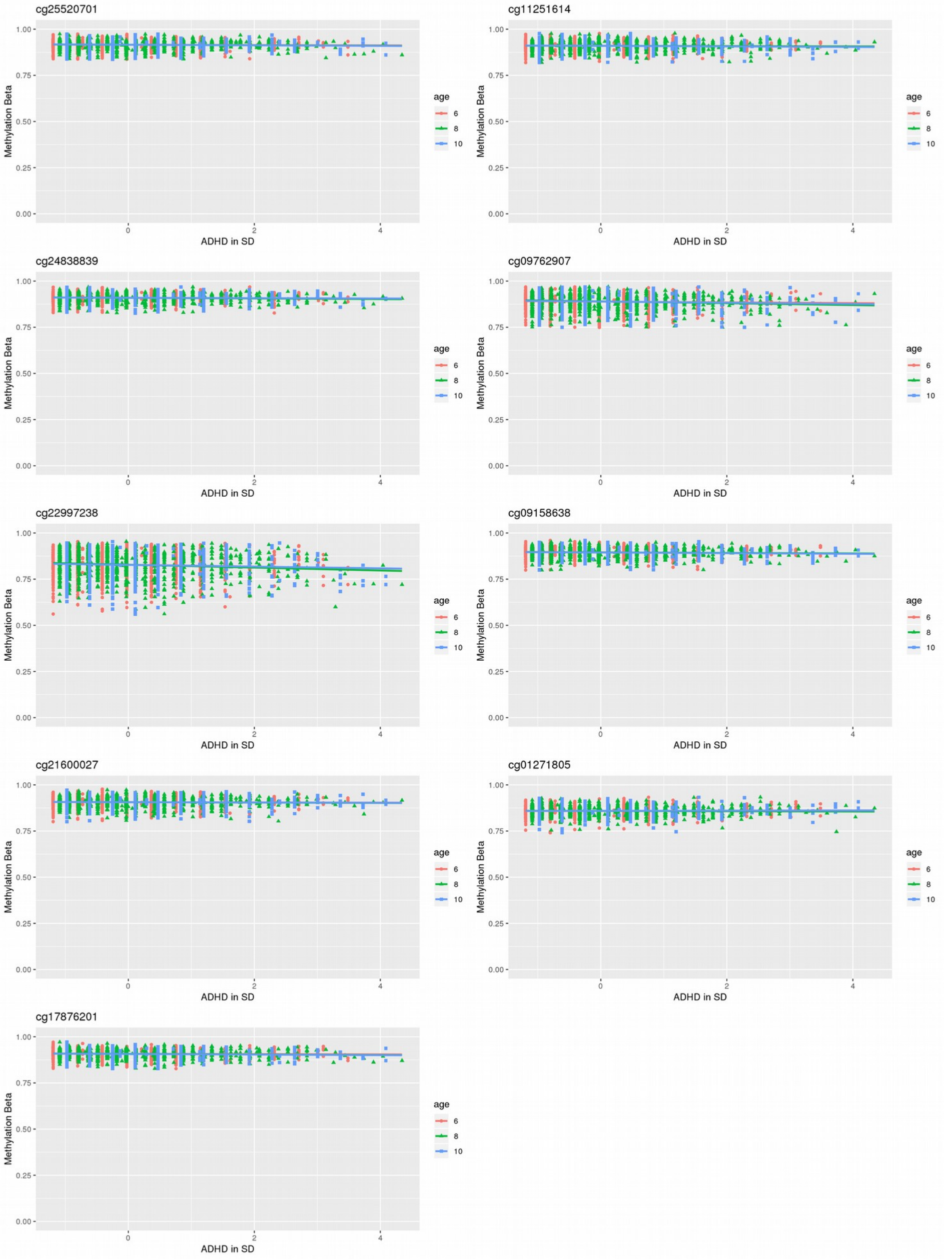

Supplement: Supplementary file 1 — Figure S1 and S2 [file 41398_2020_1058_MOESM1_ESM.pdf]
