# Supplementary material for: Association between DNA methylation and ADHD symptoms from birth to school age: a prospective meta-analysis
Source: Transl Psychiatry. 2020 Nov 12;10:398. doi: 10.1038/s41398-020-01058-z (PMC7665047; doi:10.1038/s41398-020-01058-z)
Supplement: Supplementary file 5 — Table S2-S6 [file 41398_2020_1058_MOESM5_ESM.pdf]

Supplementary Tables

Table S2: Phenotype Characteristics

Study Name	Ancestry/ Ethnicity	Instrument	EWAS	Assesment age (mean years)	Age (SD)	Total ADHD score (mean)	Total ADHD score (SD)	% Girls
ALSPAC	European	DAWBA	Birth and school-age	7.5	0.9	0.5	0.9	52
ALSPAC	European	DAWBA	Birth	10.7	0.1	0.5	0.9	51
ALSPAC	European	DAWBA	Birth	13.9	0.1	0.5	0.9	50
ALSPAC	European	DAWBA	Birth	15.4	0.2	0.5	0.9	51
GENR	European	CBCL 1.5-5	Birth and school-age	5.9	0.3	2.7	2.4	51
GENR	European	Conners	Birth	8.1	0.2	7.5	6.6	51
GENR	European	CBCL 6-18	Birth	9.7	0.3	2.6	2.8	51
GLAKU	European	CBCL	School age	12.3	0.5	1.9	2.4	54.6
HELIX	European	Conners	School-age	8.0	1.6	7.8	6.6	44.7
HELIX	Pakistani	Conners	School-age	6.6	0.2	6.7	6.9	44.3
INMA	European	Conners	Birth	6.7	0.4	7.7	6.9	52
INMA	European	CBCL 6-18	Birth	8.9	0.6	3.4	3.1	48
NEST	Black	BASC	Birth	4.8	0.5	15.8	5.4	46
NEST	White	BASC	Birth	5.0	0.6	15.6	6.5	49
PREDO	European	Conners	Birth	4.5	0.5	5.0	4.4	47.1

Table S3: Twin heritability of genome-wide significant CpG sites

CpG	A	C	E
cg01271805	0.19	0.10	0.71
cg09158638	0.00	0.06	0.94
cg09762907	0.02	0.09	0.89
cg11251614	0.00	0.07	0.93
cg17876201	0.00	0.09	0.91
cg21600027	0.00	0.04	0.96
cg22997238	0.00	0.25	0.75
cg24838839	0.15	0.00	0.85
cg25520701	0.00	0.11	0.89

Based on Hannon E, Knox O, Sugden K, Burrage J, Wong CCY, Belsky DW *et al.* Characterizing genetic and environmental influences on variable DNA methylation using monozygotic and dizygotic twins. *PLoS Genet* 2018; **14**: 1–27.

Table S4: EWAS Results for birth methylation in children with European ancestry

				Birth methylation				
CpG	Gene	Chr	Position	$n_{studies}$	n	B	SE	p
cg25520701	CREB5	7	28800657	5	2394	-3.54	0.60	4.77E-09
cg17876201	ZBTB38	3	141139991	5	2401	-4.45	1.27	6.75E-09
cg09158638	Intergenic	16	62309996	5	2414	-3.06	1.28	1.08E-08
cg21600027	Intergenic	4	124443502	5	2409	-3.10	0.84	1.90E-08
cg11251614	PPIL1	6	36839846	5	2395	-3.52	0.62	1.94E-08
cg01271805	ERC2	3	55694954	5	2413	-3.29	1.70	3.13E-08
cg24838839	Intergenic	5	61031569	5	2412	-4.06	1.88	4.10E-08
cg22997238	Intergenic	7	36014218	5	2410	-1.64	0.30	8.04E-08
cg10025904	LRRC8B	1	89996331	5	2416	-2.74	0.59	9.76E-08
cg09762907	TRERF1	6	42290256	5	2404	-2.10	0.39	1.05E-07

Chr Chromosome
$n_{studies}$ Number of studies
n Number of participants
B Regression coefficient
SE Standard error

Table S5: DNA methylation at top CpG sites regressed on prenatal stress

				Prenatal Stress				
CpG	Gene	Chr	Position	$n_{studies}$	n	B	SE	p
cg25520701	CREB5	7	28800657	1	1214	-0.18	0.85	0.84
cg17876201	ZBTB38	3	141139991	1	1214	-0.28	0.62	0.65
cg09158638	Intergenic	16	62309996	1	1214	-0.60	0.77	0.44
cg21600027	Intergenic	4	124443502	1	1214	-0.25	0.78	0.75
cg11251614	PPIL1	6	36839846	1	1214	-0.41	0.72	0.57
cg01271805	ERC2	3	55694954	1	1214	0.39	0.89	0.66
cg24838839	Intergenic	5	61031569	1	1214	-0.39	0.62	0.53
cg22997238	Intergenic	7	36014218	1	1214	-0.16	1.80	0.38
cg09762907	TRERF1	6	42290256	1	1214	-1.26	1.14	0.27

Chr Chromosome
$n_{studies}$ Number of studies
n Number of participants
B Regression coefficient
SE Standard error

Table S6: Replication of Walton et al. EWAS (ADHD trajectories and cord blood methylation)

				Discovery				Replication				
CpG	Gene	Chr	Position	$n_{studies}$	n	B	p	$n_{studies}$	n	B	SE	p
cg18587973	CDADC1	13	49822535	1	817	0.17	1.2E-06	5	1755	3.54	1.55	0.03
cg27469152	EPX	17	56282313	1	817	-0.18	2.0E-07	5	1763	-1.20	0.85	0.20
cg16290904	PEX2	8	77912348	1	817	0.17	7.4E-07	5	1761	-2.99	2.60	0.23
cg03905179	MAFK	7	1582588	1	817	0.17	1.3E-06	5	1756	0.70	1.67	0.46
cg24843380	ZNF454	5	178367827	1	817	0.17	1.6E-06	5	1762	-1.73	2.05	0.47
cg05653018	ELF3	1	201979533	1	817	0.17	1.4E-06	5	1763	0.45	0.60	0.53
cg15096815	JUN	1	59249838	1	817	-0.18	3.5E-07	5	1763	0.55	1.08	0.68
cg24481594	SKI	1	2190850	1	817	-0.20	1.5E-08	5	1763	-0.42	1.08	0.76
cg26263766	ZNF544	19	58739734	1	817	0.17	8.7E-07	5	1714	-0.31	1.13	0.84
cg01324543	CCDC30	1	42999439	1	817	-0.17	7.2E-07	5	1763	0.59	1.75	0.92
cg13714586	FBXW5	9	139838358	1	817	0.17	1.3E-06	5	1752	0.72	4.50	0.93
cg09989037	ST3GAL3	1	44300942	1	817	-0.17	9.5E-07	5	1763	-0.17	0.56	0.96
cg22193912	MAFG	17	79881523	1	817	0.17	1.3E-06	5	1763	0.24	0.81	0.99

Chr Chromosome
$n_{studies}$ Number of studies
n Number of participants
B Regression coefficient
SE Standard error

Table S7: Replication of Wilmot et al. EWAS (case-control study in school-age)

				Discovery				Replication				
CpG	Gene	Chr	Position	$n_{studies}$	n	Diff.	p	$n_{studies}$	n	B	SE	p
cg05180887	MYT1L	2	1817263	1	92	-0.05	0.04	4	2080	-0.18	0.20	0.43
cg08479516	VIPR2	7	158905536	1	92	-0.03	0.03	4	1900	0.04	0.52	0.57
cg06201514	MYT1L	2	1817409	1	92	-0.08	0.02	5	2295	-0.06	0.11	0.66
cg13444538	VIPR2	7	158905317	1	92	-0.06	0.03	4	2080	-0.06	0.23	0.74
cg10075506	MYT1L	2	1817351	1	92	-0.08	0.04	5	2295	-0.02	0.12	0.88
cg05554000	VIPR2	7	158905015	1	92	-0.05	0.02	5	2295	-0.02	0.16	0.92

Chr Chromosome
$n_{studies}$ Number of studies
n Number of participants
Dff. Difference in methylation between cases and controls (Negative values indicate hypomethylation in controls)
B Regression coefficient
SE Standard error
